# Risk factors for unsafe behaviors toward grenades among rural populations affected by explosive devices in Colombia

**DOI:** 10.1186/s13031-018-0141-5

**Published:** 2018-03-05

**Authors:** Andrew T. Boyd, Kristin Becknell, Steven Russell, Curtis Blanton, Susan T. Cookson, Oleg O. Bilukha, Mark Anderson

**Affiliations:** 1 Epidemic Intelligence Service, Centers for Disease Control and Prevention, Atlanta, GA USA; 2 Division of Global Health Protection, Center for Global Health, Centers for Disease Control and Prevention, Atlanta, GA USA; 3 Current Address: Division of Global HIV and TB, Center for Global Health, Centers for Disease Control and Prevention, Atlanta, GA USA

**Keywords:** Injury prevention, Explosive device(s), Behavioral risk, Colombia

## Abstract

**Background:**

Following decades of armed conflict, Colombia remains highly affected by explosive device (ED) contamination, especially in rural areas. Many victims are injured by EDs despite knowing their dangers. Determining risk factors for unsafe behaviors toward EDs, including grenades, is critical for preventing injuries.

**Methods:**

In 2012, CDC assisted Colombian partners in conducting a multi-stage knowledge, attitudes, and practices survey in rural ED-affected areas. Within each of 40 clusters, 28 households were selected, and participants aged 10 years or older were asked about behaviors toward EDs. Participants reported actual behaviors toward past EDs encountered and theoretical behaviors toward EDs not encountered. Behaviors were a priori classified as unsafe or safe. This analysis focuses on behaviors toward the most commonly encountered device, grenades.

**Results:**

Of 928 adult and 562 child participants, 488 (52.5%) adults and 249 (43.9%) children encountered ED, while 121 (13.7%) adults and 148 (26.9%) children received mine risk education (MRE). Among the 430 (46.7%) adults who encountered grenades, 113 (25.7%) reported unsafe behaviors; multivariable analysis showed that unsafe behavior was associated with working outdoors (adjusted odds ratio [aOR]: 1.7, 95% confidence interval [CI]: 1.1–2.7). Among the 429 (46.5%) adults who did not encounter ED, 61 (14.6%) described unsafe theoretical behaviors toward grenades; multivariable analysis showed that unsafe behavior was associated with older age (aOR: 1.02, 95% confidence limit [CL]: 1.00–1.05) and black or Afro-Colombian identity (aOR: 2.5, 95% CI 1.3–5.1). Among the 181 (32.0%) children who encountered grenades, 41 (23.8%) reported unsafe behaviors, while among the 311 (55.9%) children who did not encounter ED, 30 (10.2%) reported unsafe behavior. In both groups of children, multivariable analysis showed that unsafe behavior was associated with lower mean score on knowledge of ED, with aOR: 0.7, 95% CL: 0.6–0.9, and aOR: 0.8, 95% CL: 0.6–0.98, respectively.

**Conclusions:**

Participants reported frequent ED exposure but low receipt of MRE. Our findings should guide MRE improvement in ED-affected areas by strengthening the connection between ED knowledge and avoiding unsafe behavior, with a particular focus on people working outdoors. MRE should promote knowledge of ED risks but should also recognize socioeconomic factors that lead to engaging in unsafe behaviors.

## Background

Colombia has been in a state of internal armed conflict for decades. The Colombian government estimates that nearly 220,000 people died in the conflict between 1958 and 2012, with 81% of these deaths occurring among civilians [1]. Since 1990, multiple armed groups have used landmines, improvised explosive devices, and other types of ordnance, collectively known as explosive devices (EDs), throughout Colombia [2]. Widespread use of these devices has led to evidence of explosive device (ED) contamination in all but one of Colombia’s departments, with mapping suggesting a concentration of affectedness in rural areas [3]. The Landmine Monitor states that armed groups have used such devices “near their campsites or bases, on other paths that lead to areas of strategic importance (such as paths to main transit routes along mountain ridges)…to protect caches of explosives, weapons, medicine, and clothing….[and] in or near coca fields to prevent eradication efforts” [4]. One consequence of this widespread contamination is an elevated risk of fatal and nonfatal ED injury: Colombia had the most ED-related injuries in the world from 2005 to 2007, with a peak of over 1100 injuries in 2006 [5] and a total of 10,626 reported fatal and nonfatal injuries by the end of 2013 [6]. Though the annual number of ED injuries has decreased recently, with 368 injuries reported in 2013 [6], populations in contaminated areas remain at risk of injury.

Two interventions, explosive ordnance clearance and disposal and mine risk education (MRE), focus on reducing the risk of injury faced by populations living in ED-contaminated areas. Explosive ordnance clearance and disposal is both expensive and time-consuming, and local populations remain at risk of injury until all EDs are removed. This risk is present in Colombia, where a program to decontaminate affected areas included in the June 2016 national ceasefire accord is not expected to be completed until 2021 [7]. In areas where explosive ordnance clearance and disposal have not been conducted or are incomplete, MRE—a specific package of educational components including public awareness campaigns, trainings, and community liaison related to landmines and other types of EDs—is regarded as the best tool available for raising community awareness of the presence and dangers of EDs and for promoting safe behaviors and reducing unsafe behaviors toward EDs. MRE’s goal is to reduce the risk of both fatal and nonfatal injury [8], although evidence of effectiveness of MRE alone to reduce injury is lacking.

To better understand knowledge and perceived risk of EDs among populations in Colombia residing in ED-affected areas, and to improve targeting of MRE programming for at-risk groups, the United Nations Children’s Fund (UNICEF) Colombia, the Programa Presidencial para la Acción Integral contra Minas Antipersonal (PAICMA), the Centro Nacional de Consultoría (CNC) and the US Centers for Disease Control and Prevention (CDC) conducted a household survey of knowledge, attitudes, and practices (KAP) related to EDs in affected rural areas in 2012. For this analysis we used data from the 2012 survey to describe the extent of ED exposure and the receipt of MRE in the affected population. Additionally, we describe the potential risk factors associated with reporting unsafe behaviors toward EDs. Determining potential risk factors for unsafe behaviors toward EDs, including grenades, provides a focus for targeting MRE to specific groups at high risk and is critical for the prevention of ED injuries in affected populations.

## Methods

The survey was a cross-sectional representative assessment of adults aged 18 years or older and children aged 10 through 17 years living in ED-affected rural communities in Colombia. A target sample size of 834 households was calculated based on an assumption of 50% prevalence of exposure to EDs, a design effect of 2, and a 95% half-width confidence interval (CI) of 5%. The target sample size was inflated to 1112 households to account for an expected 25% household non-response. The final target sample size was 1120 households, comprising 40 clusters of 28 households each.

To create a sampling frame of ED-affected communities, an estimate of “ED affectedness” was created for each municipality in 31 of Colombia’s 32 departments (one was excluded due to security concerns). This estimate of a municipality’s risk of being affected by EDs was a composite of the values across six domains from 2006 to 2011: 1) the rate of ED-related fatal and non-fatal injuries per year per 10,000 rural population (from PAICMA data), 2) the rate of “dangers” (a general term that aggregated numbers of mined areas, military demining operations, unexploded ordnance, suspected dangerous areas, and suspected minefields) per year per 10,000 rural population (from PAICMA), 3) the number of combat activities over the entire period (from UN Office of Humanitarian Assistance [UNOCHA]), 4) the rate of population displacement per year per 10,000 rural population (from UNOCHA), 5) the number of hectares of illicit crops (from UN Office on Drugs and Crime [UNODC]), and 6) the number of hectares of coca crops cleared by eradication (from UNODC). To account for the different scales for each criterion, all criteria were then normalized and combined into a composite value. To ensure that surveyed municipalities were “ED-affected”, the sampling frame was then limited to the 125 municipalities with the highest estimate of ED affectedness.

Of these 125 municipalities, 24 were eliminated because of the following considerations: being declared “mine-free” (*n* = 1), containing only one ED-affected community (*n* = 2), or lacking a sufficient number of potential clusters for the substitution plan (*n* = 21). From the 101 remaining municipalities, 39 municipalities were randomly selected based on probability of selection proportional to the estimated 2009 rural population size (projected from Colombia’s 2005 census). Thirty-eight municipalities were selected once and one municipality was selected twice because of its larger population. In Colombia, each municipality consists of one *cabecera municipal*, which is the urban administrative seat, and multiple *centros poblados* (town centers), which are more rural but by definition contain at least 20 dwellings. To ensure focus on rural areas, *cabeceras municipales* were excluded, and *centros poblados* served as clusters for the survey. Within the 39 municipalities, 40 *centros poblados* were selected by simple random selection, because of a lack of population data at the *centro poblado* level. Because of security concerns at the time of the survey, 13 selected municipalities and nine *centros poblados* were replaced. Within each *centro poblado*, 28 households were selected by systematic random sampling. Finally, within each selected household, one randomly selected adult and all children aged 10 to 17 years were invited to participate. Eligible adults provided informed consent prior to participation; parents or guardians provided permission to participate for eligible children, who in turn provided assent to participate. Substitution to replace adults who could not be reached or who chose not to participate was not conducted. Colombian interviewers then administered a face-to-face adult questionnaire to participants aged 18 years and above and a slightly abbreviated child questionnaire to participants aged 10 to 17 years. Interviewers were selected by CNC from diverse regions of Colombia, and were required to have 1 year of experience in administration of household interviews in rural areas. Prior to survey administration, interviewers received a five-day training. The survey was written simultaneously in Spanish and English by CDC staff and edited by partners, and it was administered in Spanish. All survey participants were given an MRE pamphlet, including information about EDs, signs of ED contamination, and safe behaviors toward EDs, following survey completion, and representatives of selected households who chose not to participate also received a pamphlet. The survey was approved by the CDC Human Subject Research Office (CDC Protocol #6134), and it was conducted from August 10 through October 6, 2012.

The survey questionnaires included questions across five domains: demographics; media habits; knowledge, attitudes and practices related to EDs; exposure to EDs and receipt of MRE; and reporting and assistance related to ED encounters.

Behaviors related to EDs were elicited first by asking participants if they had ever encountered each of the following ED types: antipersonnel landmine, grenade, explosive booby trap, or other type of ED. If participants had encountered a device type, they were asked which behaviors they had engaged in during their most recent encounter with it (“actual behaviors”). If participants stated that they had not encountered the device type, they were asked what behaviors they would engage in were they to encounter it (“theoretical behaviors”). Both actual and theoretical behaviors were a priori classified as “unsafe”, “safe”, or “neither unsafe nor safe”. Classification of behaviors was done by adapting a list of behaviors used in MRE materials in Colombia and elsewhere to the Colombian context. Unsafe behaviors included approaching or moving the ED, while safe behaviors included telling an authority or leaving the affected area. Behaviors classified as neither unsafe nor safe included observing the ED without approaching or touching it (Table 1).

**Table 1:** Unsafe, safe, and neither unsafe nor safe behaviors toward explosive devices (EDs) among participants in a household knowledge, attitudes, and practices (KAP) survey—Colombia, 2012

UNSAFE BEHAVIORS
Placed some kind of sign to warn the community (like a bundle of straw or a branch cross)
Ignored the device (except in cases where participant ignored it because the military had it)
Did not leave
Did not tell an authority or another person
Approached the device
Played with the device
Threw salt at the device
Threw something else at the device
Opened the device
Picked the device up
Disactivated the device
Hit the device with a stick or another object
Moved the device
Kicked the device
Stepped on the device
Burned the device
Threw the device in water
Touched the device or handled it but did not move it
SAFE BEHAVIORS
Told an authority or another person
Left
Returned trying to follow his/her tracks
Did not play with the device
Did not throw salt at the device
Did not throw other things at the device
Did not open the device
Did not pick the device up
Did not hit the device with a stick or other object
Did not move the device
Did not kick the device
Did not step on the device
Did not burn the device
Did not throw the device in water
Did not touch or handle the device
Did not approach the device
NEITHER SAFE NOR UNSAFE BEHAVIORS
Ignored the device because the military had it
Took a look but did not touch or handle the device
Did not take a look but did not touch or handle the device
Did not place some kind of sign to warn the community (like a bundle of straw or a branch cross)
Did not return trying to follow his/her tracks
Other

For this analysis, a participant who reported engaging in any unsafe behavior toward a device was placed in the “unsafe behavior” category regardless of whether s/he also engaged in safe behaviors with that device. Because each participant was asked about behaviors toward each of the four types of ED, it was possible that a participant could have reported only “safe” behavior toward one device but “unsafe” behavior toward another. Thus, single assignment of a participant to an “unsafe” or “safe” category was impossible. In this analysis, we focused on actual and theoretical behaviors toward only the most commonly encountered device, grenades. All participants who had not encountered a grenade were asked about theoretical behaviors toward grenades, but that group included both participants who had not encountered any ED and those who had not encountered a grenade but had encountered other ED types. In this analysis we focused on theoretical behaviors toward grenades only among the subpopulation who had not encountered any ED.

Data were analyzed using complex survey procedures in SAS version 9.4 [9] to take into account sample weighting, clustering, and stratification. First, bivariate analyses were conducted for both actual and theoretical behaviors, with a binary outcome of reporting any unsafe behavior toward grenades and reporting no unsafe behavior toward grenades. Covariates to be included were selected from among the domains of the questionnaire, and were limited to those that described a unique participant characteristic. We used the following covariates for these analyses:

1. Demographics: age, sex, race/ethnicity, highest level of education reached, location of occupation, and displacement;
2. Media habits: number of media sources consumed;
3. Knowledge, attitudes, and practices related to EDs: knowledge of EDs and perceived community ED risk; and
4. Exposure to EDs and receipt of MRE: number of encounters with all types of ED (for actual unsafe behavior only) and receipt of MRE.

Race/ethnicity was self-identified and was asked only of adults. Level of education reached was defined as “low” if the participant had not advanced beyond primary school and “high” if the participant reported any education beyond primary school. Location of occupation was categorized as either “outdoor occupation” (including occupations in fields, forests, water, construction, mines, and roads) or “indoor occupation” (including occupations in markets, shops, restaurants, schools, offices, health centers, and homes). We assessed displacement by asking each participant how s/he came to live in his/her community, noting whether his/her resettlement included displacement. We determined the number of media sources consumed by summing the total number of media sources used in a typical week among newspapers, radio, television, and the internet. Knowledge of EDs was assessed by the number of correct responses to eight true/false statements about ED (Table 2). We assessed perceived community ED risk with the following two questions: “Have you ever heard of accidents in which EDs have exploded in this community and caused human wounds or deaths?”, asked of both adults and children, and “Is your community affected by the presence of EDs at this time?”, asked of adults only.

**Table 2:** True/false statements used to assess ED knowledge among participants in a household KAP survey—Colombia, 2012

1) If you find a grenade you can take it home and keep it, because if it did not explode when it was used, it is defective and will not cause harm.
2) If it is raining, it is safe to take shelter in an abandoned house.
3) Throwing rocks at an antipersonnel mine makes it explode without anyone being injured.
4) Some mines are made of plastic.
5) Some mines are hung in trees.
6) Walking gently and carefully through a suspected minefield will ensure that you get out without causing any mines to explode.
7) Driving a herd or group of animals through a field that is suspected to be mined renders it safe to cross.
8) Combat occurred in a field 6 months ago. It is safe to pass through the field without major risk because a lot of time has passed.

To model the associations between variables and adjust for potential confounding among covariates, we constructed two multivariable logistic regression models of risk factors for actual unsafe behavior, one for adults and one for children. The models were constructed using backward elimination [10]; all covariates listed above were included as initial candidates for the full model. At each step in the process, we conducted hypothesis testing to assess the significance of each regression coefficient. Then we manually removed the covariate that produced the regression coefficient with the largest *p* value. As a criterion for inclusion in the final model, we specified an alpha level of .05 to ensure that each regression coefficient was significantly different from zero. In each model, age was included as a continuous variable. To assess the validity of this assumption, the relationship between age and unsafe behavior was examined by categorizing age groups and plotting those age groups (on the x-axis) with the log odds of unsafe behavior (on the y-axis). Based on these plots, we concluded the assumption of linearity was reasonable. The process of multivariable model construction was then repeated with theoretical unsafe behavior as the outcome.

## Results

The final sample size was 1101 households, and there was an 88% household response rate. In 969 households, a total of 1490 individuals—928 adults and 562 children—participated in the survey. Among both adult and child participants, sex was equally distributed (Table 3). Among adults, the mean age was 41.5 years and the majority (71.4%) self-identified as Mestizo or white. The mean age of child participants was 13.1 years. Among adult participants, 38.4% had an outdoor occupation, and displacement had been a resettlement factor for 9.2% of participants. Assessing ED knowledge, both adult and child participants had a mean of 5.4 correct answers out of eight true/false statements about ED. For perceived community ED risk, 25.9% of adults and 22.1% of children had heard of ED accidents leading to injury or death, and 13.3% of adults believed their community to be affected by EDs at the time of the survey. A total of 52.5% of adults and 43.9% of children had encountered ED, while only 13.7% of adults and 26.9% of children had received MRE.

**Table 3:** Demographic and explosive device (ED) exposure characteristics of survey participants in a household KAP survey—Colombia, 2012

Characteristic^a^	Adults (*N* = 928)		Children (*N* = 562)	
*Demographics*	*n* (%)^b^	95% CI	*n* (%)^b^	95% CI
Male	451 (49.3)	46.6–52.0	290 (51.7)	47.1–56.2
Female	477 (50.7)	48.0–53.4	272 (48.3)	43.8–52.9
Mean age in years (95% CL)	41.5	40.3–42.8	13.1	12.9–13.3
Race/Ethnicity^c^			*Not asked*	
Indigenous	123 (15.0)	9.3–23.3		
Black or Afro-Colombian	121 (13.6)	8.4–21.3		
Mestizo or White	636 (71.4)	61.7–79.5		
No education beyond primary school	598 (62.7)	57.9–67.3	272 (48.7)	42.0–55.5
Outdoor occupation	354 (38.4)	34.8–42.1	37 (6.6)	4.5–9.5
Displaced	89 (9.3)	6.4–13.3	*Not asked*	
*Media habits*				
Mean number of media sources used per week (newspaper, radio, TV, internet) (95% CL)	2.2	2.1–2.3	2.8	2.7–2.9
*Knowledge of EDs*				
Mean number correct out of 8 true/false statements about ED (95% CL)	5.4	5.2–5.6	5.4	5.2–5.7
*Perceived community ED risk*				
Has ever heard of accidents in which EDs have exploded in the community and caused humaninjury or death	249 (25.9)	17.7–36.1	127 (22.1)	14.9–31.5
Believes community is affected by the presence of ED at this time	129 (13.3)	7.7–22.1	*Not asked*	
*Exposure to EDs and receipt of MRE*				
Has encountered any ED	488 (52.5)	47.4–57.6	249 (43.9)	38.7–49.2
Has encountered a grenade	430 (46.7)	41.4–52.2	181 (32.0)	27.1–37.2
Has encountered ED on more than one occasion	194 (21.0)	17.3–25.2	87 (15.5)	11.8–20.2
Has received MRE	121 (13.7)	10.2–18.2	148 (26.9)	20.7–34.1

^a^Means are presented with 95% confidence limits (CL)

^b^Percentages are weighted based on probability of selection at each stage

^c^48 adults did not specify ethnicity/race

Among the 429 adults who had encountered grenades and reported their behaviors during those encounters, 113 (25.7%) reported actual unsafe behaviors toward grenades (Table 4). Bivariate analysis found that the odds of a male reporting unsafe behavior was 1.8 times (95% confidence interval [CI]: 1.1–2.7) the odds that a female reported unsafe behavior. Additionally, the odds that a participant with an outside occupation reported unsafe behavior was 1.7 times (95% CI: 1.1–2.7) those of a participant who did not have an outside occupation reporting unsafe behavior. In multivariable analysis, only having an outside occupation remained significant. Though those who had received MRE had 1.6 times the odds of reporting unsafe behavior compared with those who had not received MRE, this result was not statistically significant. There was no difference in the odds of reporting actual unsafe behavior in other examined demographic characteristics, media habits, ED knowledge, or perceived risk of ED (Table 4).

**Table 4:** Factors associated with actual unsafe behavior toward grenades among adults exposed to grenades (*N* = 429)^a^ in a household KAP survey—Colombia, 2012

Characteristic^b^	Reported unsafe behavior		Did not report unsafe behavior		Odds ratio from bivariate analysis (95% CI)	Odds ratio from multivariable analysis (95% CI)
	*n* (%)^c^	95% CI	*n* (%)^c^	95% CI		
Total	113(25.7)	20.2–32.1	316(74.3)	67.9–79.8	*n/a*	*n/a*
*Demographics*						
Male	79 (29.3)	22.7–37.0	189(70.7)	63.0–77.3	**1.8 (1.1–2.7)** ^d^	
Female	34 (19.0)	13.2–26.8	127(81.0)	73.2–86.8	*Ref*	
Mean age in years (95% CL)	39.5	36.5–42.4	38.2	36.5–40.0	1.0 (0.99–1.02)	
Race/ethnicity						
Indigenous	14 (23.7)	14.1–37.1	49 (76.3)	62.9–86.0	1.0 (0.5–2.0)	
Black or Afro-Colombian	19 (36.7)	23.9–51.6	31 (63.3)	48.4–76.1	1.8 (0.9–3.5)	
Mestizo or white	75 (24.2)	18.2–31.4	224(75.8)	68.6–81.8	*Ref*	
Education beyond primary school	48 (26.7)	18.9–36.2	134(73.3)	63.8–81.1	1.1 (0.7–1.8)	
No education beyond primary school	65 (25.0)	18.9–32.2	182(75.0)	67.8–81.1	*Ref*	
Outdoor occupation	64 (30.7)	23.5–39.1	145(69.3)	60.9–76.5	**1.7 (1.1–2.7)**	**1.7 (1.1–2.7)**
Not outdoor occupation	49 (20.7)	14.8–28.3	170(79.3)	71.7–85.2	*Ref*	
Displaced	9 (21.2)	10.6–37.7	34 (78.8)	62.3–89.4	0.8 (0.3–1.9)	
Not displaced	104(26.2)	20.2–33.3	281(73.8)	66.7–79.8	*Ref*	
*Media habits*						
Mean number of media sources used per week (newspaper, radio, TV, internet) (95% CL)	2.3	2.1–2.5	2.3	2.2–2.5	0.9 (0.7–1.3)	
*Knowledge of EDs*						
Mean number correct out of 8 true/false statements about ED (95% CL)	5.6	5.3–6.0	5.8	5.7–6.0	0.9 (0.8–1.1)	
*Perceived community ED risk*						
Has ever heard of accidents in which EDs have exploded in the community and caused human injury or death	40 (27.3)	20.7–35.0	109(72.7)	65.0–79.3	1.1 (0.7–1.8)	
Has not heard of accidents	72 (24.9)	18.4–32.9	205(75.1)	67.1–81.6	*Ref*	
Believes community is affected by the presence of ED at this time	16 (24.0)	16.0–34.2	62 (76.0)	65.8–84.0	0.9 (0.5–1.5)	
Does not believe community is affected	97 (26.2)	20.3–33.1	253(73.8)	67.0–79.7	*Ref*	
*Exposure to EDs and receipt of MRE*						
Has encountered ED on one occasion	53 (22.1)	15.4–30.7	172(77.9)	69.3–84.6	*Ref*	
Has encountered ED on 2–3 occasions	19 (21.1)	13.5–31.4	76 (78.9)	68.6–86.5	0.9 (0.5–1.7)	
Has encountered ED on 4 or more occasions	29 (37.0)	24.9–51.0	49 (63.0)	49.0–75.1	2.1 (1.0–4.2)	
Has received MRE	23 (33.3)	23.3–45.0	56 (66.7)	55.0–76.7	1.6 (0.9–2.8)	
Has not received MRE	90 (23.8)	18.0–30.9	260(76.2)	69.1–82.0	*Ref*	

^a^One participant who had encountered a grenade but reported no behaviors toward it was excluded from this analysis

^b^Means are presented with 95% confidence limits (CL)

^c^Percentages are weighted based on probability of selection at each stage

^d^Bold text signifies statistical significance at *p* < 0.05

Among 180 children who had encountered grenades and reported their behaviors during those encounters, 41 (23.8%) reported actual unsafe behaviors toward grenades (Table 5). Bivariate analysis showed that a higher mean number of correct answers about EDs was associated with a reduced odds of reporting unsafe behavior, with an odds ratio (OR) of reporting unsafe behavior of 0.7 (95% confidence limit [CL]: 0.6–0.9) for each increase of one in the mean number correct out of eight true/false statements about EDs. Multivariable analysis showed no additional significant associations between examined covariates and reporting unsafe behavior, and thus ED knowledge was the only covariate retained, with an OR identical to that from bivariate analysis. There was no difference in the odds of reporting actual unsafe behavior in examined demographic characteristics, media habits, perceived risk of EDs, number of exposures to EDs, or receipt of MRE (Table 5).

**Table 5:** Factors associated with reported unsafe behavior toward grenades among children exposed to grenades (*N* = 180)^a^ in a household KAP survey—Colombia, 2012

Characteristic^b^	Reported unsafe behavior		Did not report unsafe behavior		Odds ratio from bivariate analysis (95% CI)
	n (%)^c^	95% CI	n (%)^c^	95% CI	
Total	41 (23.8)	17.4–31.7	139(76.2)	68.3–82.6	*n/a*
*Demographics*					
Male	28 (24.7)	17.1–34.3	91 (75.3)	65.7–82.9	1.2 (0.5–2.5)
Female	13 (22.1)	12.7–35.5	48 (77.9)	64.5–87.3	*Ref*
Mean age in years (95% CL)	14.0	13.3–14.8	13.8	13.5–14.1	1.1 (0.9–1.2)
In school	32 (23.0)	16.3–31.4	113(77.0)	68.6–83.7	0.8 (0.4–1.7)
Not in school	9 (27.2)	15.4–43.5	26 (72.8)	56.5–84.6	*Ref*
Outdoor occupation	5 (25.6)	9.0–54.4	17 (74.4)	45.6–91.0	1.2 (0.3–4.2)
Not outdoor occupation	33 (23.0)	16.5–31.0	115(77.0)	69.0–83.5	*Ref*
*Media habits*					
Mean number of media sources used per week (newspaper, radio, TV, internet) (95% CL)	3.1	2.8–3.3	2.8	2.7–3.0	1.4 (0.8–2.3)
*Knowledge of EDs*					
Mean number correct out of 8 true/false statements about ED (95% CL)	4.9	4.3–5.5	5.8	5.5–6.2	**0.7 (0.6–0.9)** ^d^
*Perceived community ED risk*					
Has ever heard of accidents in which EDs have exploded in the community and caused human injury or death	10 (17.3)	9.2–30.1	47 (82.7)	69.9–90.8	0.6 (0.2–1.5)
Has not heard of accidents	31 (27.0)	18.2–38.0	91 (73.0)	62.0–81.8	*Ref*
*Exposure to EDs and receipt of MRE*					
Has encountered ED on one occasion	24 (25.4)	16.3–37.3	76 (74.6)	62.7–83.7	*Ref*
Has encountered ED on 2–3 occasions	10 (20.9)	11.2–35.7	41 (79.1)	64.3–88.8	0.8 (0.3–2.1)
Has encountered ED on 4 or more occasions	6 (28.3)	11.9–53.6	15 (71.7)	46.4–88.1	1.2 (0.4–3.2)
Has received MRE	14 (24.5)	13.5–40.3	48 (75.5)	59.7–86.5	1.0 (0.5–2.4)
Has not received MRE	27 (23.9)	16.8–32.7	89 (76.1)	67.3–83.2	*Ref*

^a^One participant who had encountered a grenade but reported no behaviors toward it was excluded from this analysis

^b^Means are presented with 95% confidence limits (CL)

^c^Percentages are weighted based on probability of selection at each stage

^d^Bold text signifies statistical significance at *p* < 0.05

Among 428 adults who had not encountered ED and reported theoretical behaviors, 61 (14.6%) described unsafe theoretical behaviors toward grenades (Table 6). Results of bivariate and multivariable analyses showed that increasing age was associated with unsafe theoretical behavior, with an OR of 1.02 (95% CL: 1.00–1.05) for every 1 year increase in age. Additionally, participants who self-identified as black or Afro-Colombian had 2.5 (95% CI: 1.3–5.1) times greater odds of describing unsafe theoretical behaviors compared with participants who self-identified as Mestizo or white. Those who self-identified as indigenous also had 2.3 times greater odds of unsafe theoretical behavior compared with participants who self-identified as Mestizo or white, but this finding did not achieve statistical significance, possibly due to the small sample size of this ethnic group. There was no detected difference in the odds of theoretical unsafe behavior in other examined demographic characteristics, media habits, perceived risk of EDs, or receipt of MRE (Table 6).

**Table 6:** Factors associated with theoretical unsafe behavior toward grenades among adults who have seen no explosive devices (ED) (*N* = 428)^a^ in a household KAP survey—Colombia, 2012

Characteristic^b^	Described unsafe behavior		Did not describe unsafe behavior		Odds ratio from bivariate analysis (95% CI)	Odds ratio from multivariable analysis (95% CI)
	n (%)^c^	95% CI	n (%)^c^	95% CI		
Total	61 (14.6)	11.3–18.7	367(85.4)	81.3–88.7	*n/a*	*n/a*
*Demographics*						
Male	23 (15.0)	9.6–22.5	126(85.0)	77.5–90.4	1.1 (0.6–1.9)	
Female	38 (14.4)	10.6–19.3	241(85.6)	80.7–89.4	*Ref*	
Mean age in years (95% CL)	50.0	45.0–55.1	43.9	42.0–45.8	**1.02 (1.00–1.05)** ^d^	**1.02 (1.00–1.05)**
Race/ethnicity						
Indigenous	9 (23.0)	10.5–43.4	37 (77.0)	56.6–89.5	2.3 (0.8–6.4)	2.3 (0.9–6.3)
Black or Afro-Colombian	14 (24.9)	16.5–35.7	52 (75.1)	64.3–83.5	**2.5 (1.2–5.2)**	**2.5 (1.3–5.1)**
Mestizo or white	37 (11.6)	8.1–16.3	251(88.4)	83.7–91.9	*Ref*	*Ref*
Education beyond primary school	46 (16.3)	12.3–21.3	254(83.7)	78.7–88.0	1.6 (0.8–3.3)	
No education beyond primary school	15 (10.7)	5.9–18.6	113(89.3)	81.4–94.1	*Ref*	
Outdoor occupation	16 (13.3)	7.3–23.3	103(86.7)	76.7–92.7	1.2 (0.6–2.4)	
Not outdoor occupation	45 (15.0)	11.5–19.5	264(85.0)	80.5–88.5	*Ref*	
Displaced	6 (15.9)	7.4–30.8	33 (84.1)	69.2–92.6	1.1 (0.5–2.6)	
Not displaced	55 (14.5)	11.2–18.5	334(85.5)	81.5–88.8	*Ref*	
*Media habits*						
Mean number of media sources used per week (newspaper, radio, TV, internet) (95% CL)	2.1	1.8–2.3	2.2	2.0–2.3	0.8 (0.5–1.3)	
*Knowledge of EDs*						
Mean number correct out of 8 true/false statements about ED (95% CL)	4.9	4.5–5.3	5.1	4.9–5.3	0.9 (0.8–1.1)	
*Perceived community ED risk*						
Has ever heard of accidents in which EDs have exploded in the community and caused human injury or death	14 (16.0)	9.0–27.0	67 (84.0)	73.0–91.0	1.2 (0.6–2.5)	
Has not heard of accidents	46 (13.8)	10.2–18.6	299(86.2)	81.4–89.8	*Ref*	
Believes community is affected by the presence of ED at this time	5 (10.7)	3.8–26.6	35 (89.3)	73.4–96.2	0.7 (0.2–2.2)	
Does not believe community is affected	55 (14.5)	11.1–18.8	332(85.5)	81.2–88.9	*Ref*	
*Receipt of MRE*						
Has received MRE	2 (5.4)	1.2–21.6	31 (94.6)	78.4–98.8	0.3 (0.07–1.5)	
Has not received MRE	59 (15.4)	12.0–19.6	336(84.6)	80.4–88.0	*Ref*	

^a^One participant who had not encountered ED reported no theoretical behavior, so was excluded from this analysis

^b^Means are presented with 95% confidence limits (CL)

^c^Percentages are weighted based on probability of selection at each stage

^d^Bold text signifies statistical significance at *p* < 0.05

Among 311 children who had not encountered ED, 30 (10.2%) described unsafe theoretical behaviors toward grenades (Table 7). Results of bivariate analysis showed that a higher ED knowledge mean score was associated with a reduced odds of unsafe theoretical behavior, with an OR of 0.8 (95% CL: 0.60–0.98) for each increase of one in the mean number correct out of eight true/false statements about EDs. Multivariable analysis showed no additional significant associations between examined covariates and reporting unsafe behavior, and thus ED knowledge was the only covariate retained, with an OR identical to that from bivariate analysis. There was no difference in odds of unsafe theoretical behavior in examined demographic characteristics, media habits, perceived risk of EDs, or receipt of MRE (Table 7).

**Table 7:** Factors associated with theoretical unsafe behavior toward grenades among children who have seen no explosive devices (ED) (*N* = 311) in a household KAP survey—Colombia, 2012

Characteristic^a^	Described unsafe behavior		Did not describe unsafe behavior		Odds ratio from bivariable analysis (95% CI)
	n (%)^b^	95% CI	n (%)^b^	95% CI	
Total	30 (10.2)	7.2–14.2	281 (89.8)	85.8–92.8	*n/a*
*Demographics*					
Male	13 (10.9)	6.7–17.2	117 (89.1)	82.8–93.3	1.1 (0.6–2.1)
Female	17 (9.7)	6.4–14.3	164 (90.3)	85.7–93.6	*Ref*
Mean age in years (95% CL)	12.4	11.7–13.1	12.9	12.6–13.1	0.9 (0.8–1.1)
In school	25 (9.6)	6.2–14.5	251 (90.4)	85.5–93.8	0.6 (0.2–2.0)
Not in school	5 (15.0)	6.1–32.3	30 (85.0)	67.7–93.9	*Ref*
Outdoor occupation	0 (0)	*n/a*	10 (100)	*n/a*	Cannot calculate
Not outdoor occupation	30 (10.6)	7.6–14.7	268 (89.4)	85.3–92.4	
*Media habits*					
Mean number of media sources used per week (newspaper, radio, TV, internet) (95% CL)	2.1	1.8–2.3	2.2	2.0–2.3	0.8 (0.6–1.3)
*Knowledge of ED*					
Mean number correct out of 8 true/false statements about ED (95% CL)	4.5	3.6–5.3	5.3	5.0–5.6	**0.8 (0.6–0.98)** ^c^
*Perceived community ED risk*					
Has ever heard of accidents in which EDs have exploded in the community and caused human injury or death	5 (9.4)	3.8–21.2	52 (90.6)	78.8–96.1	0.9 (0.4–2.2)
Has not heard of accidents	25 (10.4)	7.6–14.1	228 (89.6)	85.9–92.4	*Ref*
*Receipt of MRE*					
Has received MRE	4 (6.3)	2.3–16.0	63 (93.7)	84.0–97.7	0.5 (0.2–1.5)
Has not received MRE	26 (11.4)	8.1–15.8	215 (88.6)	84.2–91.9	*Ref*

^a^Means are presented with 95% confidence limits (CL)

^b^Percentages are weighted based on probability of selection at each stage

^c^Bold text signifies statistical significance at *p* < 0.05

## Discussion

Several of the findings on ED exposure and perceived risk are concerning. ED exposure was relatively common in the survey population: 53% of adults and 44% of children surveyed reported having encountered ED, with nearly 50% of adults and over 30% of children reporting exposure to grenades. Despite this widespread exposure, relatively few people—only 14% of adults and 27% of children—had ever received MRE. Additionally, the population’s perception of risk was comparatively low: only 13% of adults felt that their communities were currently affected by EDs. Thus, in this population there may be a disconnect between high population exposure to ED and lower perceived risk.

Among adults who had encountered grenades, reporting of actual unsafe behavior toward grenades was associated in bivariate analysis with being male and working outdoors, though only working outdoors remained significant in multivariable analysis. The two potential risk factors identified in bivariate analysis are consistent with those found in other settings. In Cambodia, males represented 88% of all reported fatal and non-fatal ED injuries, and the majority of these incidents happened while the victims were pursuing livelihood activities outdoors [11]. In Afghanistan, analysis of ED injury surveillance data showed that 92% of injuries occurred among males, and that one of the most common risk factors for injury was engaging in outdoor occupations and livelihood activities, including farming, tending animals, and gathering food and water [12, 13]. Higher rates of injury among males compared with females were found in reviews of ED injury surveillance data in Chechnya and Nepal [14, 15]. It may be that surveyed males in Colombia engage in unsafe behaviors because of a real or perceived need to conduct livelihood activities or to access economic or social resources.

Among adults who had not encountered any ED, describing theoretical unsafe behavior was associated with older age and self-described black or Afro-Colombian identity, and both of these risk factors remained significant in multivariable analysis. The magnitude of the association between increasing age and unsafe behavior was small, with an odds ratio of 1.02 for a one-year increase in age, though across greater age differences, it becomes more meaningful. For instance, someone 40 years old would have 1.22 times greater odds of unsafe behavior than someone 30 years old. Those of self-described Afro-Colombian identity had 2.5 times greater odds of unsafe behavior compared with those of self-described Mestizo or white identity. Neither older age nor minority ethnicity has been previously described as being associated with unsafe behavior toward EDs, and neither were significantly associated with reported actual behavior during the most recent ED encounter. It may be that these groups are less likely to receive messaging about safe behaviors toward EDs.

Among children, regardless of whether they had been exposed to grenades or to no ED, greater knowledge of ED risks, as measured by higher knowledge scores, was associated with a mildly decreased risk of engaging in both actual and theoretical unsafe behavior. In contrast, receipt of MRE was not significantly associated with a decreased risk of engaging in unsafe behavior. Though receipt of MRE in and of itself was not associated with less unsafe behavior toward grenades, MRE intended to reduce engagement in unsafe behaviors may still be useful when targeted to children if it increases ED knowledge or emphasizes the risks of ED contamination.

In contrast with this finding in children, higher scores among both groups of adults—those who had been exposed to grenades or to no ED—were not associated with a decreased risk of actually or theoretically engaging in unsafe behavior. Thus, surveyed adults who know the dangers of EDs may still engage in risky behavior toward grenades. This finding is well documented in the literature [16]. While the most documented risk factor for unsafe behavior is engaging in livelihood or economic activities, broader social or environmental factors may play a role in informed people’s deciding to engage in unsafe behavior toward ED. One example of the complexity of motivation to engage in unsafe behavior toward ED was documented in Cambodia, where villagers undertook their own demining operations without proper training or equipment [11, 17]. There, adult villagers reported handling EDs because of inadequate or nonexistent alternative demining operations, a desire to protect children, an opportunity to extract economic value from EDs, or episodic increased economic desperation (e.g., when someone needed to buy medicine for a sick family member) [17]. In Lao People’s Democratic Republic (PDR), villagers perceived reduced risk of unsafe behavior if they had handled EDs without injury in the past [16]. Thus, MRE may be more useful to adults in ED-contaminated areas when it recognizes and addresses the competing concerns of physical safety versus socioeconomic pressures.

Among both adults and children, a higher proportion of people reported actual unsafe behavior toward grenades compared with people who described theoretical unsafe behavior toward grenades. This discrepancy highlights a possible disconnect between knowledge of safe and unsafe behaviors upon exposure to EDs and actual engagement in such behaviors—in other words, between what people say they would do and what they actually do. Further research on the divergence between theoretical and actual behaviors could lead to the improvement of MRE content and other mine action initiatives.

An unexpected finding was that among both adults and children in the surveyed population, receipt of MRE was not associated with decreased odds of actual or theoretical unsafe behavior toward grenades. In fact, among adults who had encountered grenades, those who had received MRE had a moderately increased odds of reporting unsafe behavior compared with those who had not received MRE, although this result was not statistically significant. The lack of association between receipt of MRE and safe behavior is not unique to our survey. MRE, as delivered in many ED-affected countries in the world, provides information on the dangers of EDs and lists “dos and don’ts” for ED interactions in local environments [16, 17]. In Lao PDR, a KAP study conducted both before and after an MRE program in ED-affected areas found improved community knowledge of ED risks and unsafe behavior, but persistent unsafe behaviors [16]. In a review of Afghan ED injury surveillance data, those injured who had received MRE were much more likely to report awareness of being in an ED-contaminated area, yet they still entered such areas and were injured [18]. Similarly, in a KAP survey toward EDs conducted in 2015 among Syrian refugees in Turkey, 15% of adults and 10% of children reported entering an area suspected to contain ED, despite knowing the dangers of entering [19]. It has been hypothesized that MRE that incorporates a more holistic understanding of the social and environmental pressures to engage unsafely with EDs would be more effective in reducing ED injury [16, 20]. Additionally, seeking community input to establish a framework of acceptable and unacceptable behavior, adapted to the local context, may improve the efficacy of MRE in reducing injury [17, 21].

The results of this analysis are subject to several limitations. First, our analysis focused on unsafe behavior upon exposure to only one type of ED (grenades) because these were the devices most frequently encountered by survey participants. However, this decision limits the generalizability of the findings, because behavioral risk factors associated with grenade exposure may not be the same as those associated with exposure to landmines, explosive booby traps, or other ED types. Second, our analysis did not examine differences in behavior by type of employment or by prior military experience, and current or former armed forces members may approach grenades differently from the general public. Third, security concerns led to the replacement of 13 municipalities and nine *centros poblados* initially selected for this study. Because the replaced areas may have had ED contamination, their exclusion may have affected the survey results. Fourth, this analysis did not explore the reasons given by participants for engaging in unsafe behaviors toward grenades, and this lack of information may limit the ability of MRE organizations to craft targeted preventive behavioral interventions. Fifth, since this analysis was conducted using data from a cross-sectional survey, it could not capture temporal relationships between receipt of MRE and reported behaviors. In other words, reported actual behaviors toward EDs may have occurred before or after receipt of MRE. Therefore, this analysis could not show any direct impact of MRE on actual behavior toward ED. Finally, survey results relied on what participants self-reported as behavior toward EDs, and participants may have underreported unsafe behaviors due to tendencies to answer questions either in a socially desirable way or in a manner that would avoid shaming or blaming community members for engaging in unsafe behaviors [17, 22].

## Conclusions

This analysis provides valuable insights into many aspects of ED exposure, knowledge, and behavior in affected rural communities in Colombia. Notably, ED exposure was common, but perceived ED risk was low, and receipt of MRE uncommon. While higher ED knowledge was associated with decreased reporting of unsafe behaviors among children who had encountered a grenade and among children who had not encountered EDs, this association was not seen among adults. Receipt of MRE was not found to be associated with decreased reporting of unsafe behaviors among adults or children.

This analysis provides several suggestions for MRE programs in Colombia. First, MRE in general should continue among this highly exposed population, largely because it is the only tool available to address and potentially mitigate the risk of injury in contaminated areas where explosive ordnance clearance and disposal are unavailable or incomplete. However, MRE content and delivery methods in Colombia should be reviewed in light of the finding that receipt of MRE was not associated with a decrease in unsafe actual or theoretical behavior among either adults or children. Specifically, it should seek to connect ED knowledge with how to avoid unsafe behaviors, with a particular focus on males and people working outdoors. Third, among adults and children, MRE should promote knowledge of ED risks, but should also recognize broader social and economic factors shaping the willingness to engage in unsafe behaviors. Fourth, MRE materials must be developed with input from affected communities to improve content and delivery methods. This community involvement could also provide insights into bridging context-specific gaps between what people report they would do and what they actually do when encountering EDs. Ultimately, ongoing use of MRE as an intervention to reduce unsafe behavior toward EDs among this population requires further evaluation of its direct effects on behavior. Such an evaluation may allow Colombian partners to more effectively reduce the risk of ED injury in the rural population of Colombia, who continue to be exposed to EDs even as the country moves toward a sustained peace.

## Abbreviations

CDC: (United States) Centers for disease control and prevention
CI: Confidence interval
CL: Confidence limit
CNC: Centro Nacional de Consultoría
ED: Explosive device
KAP: Knowledge, attitudes and practices
MRE: Mine risk education
PAICMA: Programa Presidencial para la Acción Integral contra Minas Antipersonal
UNICEF: United Nations Children’s Fund
